# Partial least square regression applied to the QTLMAS 2010 dataset

**DOI:** 10.1186/1753-6561-5-S3-S7

**Published:** 2011-05-27

**Authors:** Albart Coster, Mario P L Calus

**Affiliations:** 1 Animal Breeding and Genomics Centre, Wageningen University, Wageningen, The Netherlands; 2 Animal Breeding and Genomics Centre, Animal Science Group, Lelystad, The Netherlands

## Abstract

**Background:**

Partial least square regression (PLSR) was used to analyze the data of the QTLMAS 2010 workshop to identify genomic regions affecting either one of the two traits and to estimate breeding values. PLSR was appropriate for these data because it enabled to simultaneously fit several traits to the markers.

**Results:**

A preliminary analysis showed phenotypic and genetic correlations between the two traits. Consequently, the data were analyzed jointly in a PLSR model for each chromosome independently. Regression coefficients for the markers were used to calculate the variance of each marker and inference of quantitative trait loci (QTL) was based on local maxima of a smoothed line traced through these variances. In this way, 25 QTL for the continuous trait and 22 for the discrete trait were found. There was evidence for pleiotropic QTL on chromosome 1. The 2000 most important markers were fitted in a second PLSR model to calculate breeding values of the individuals. The accuracies of these estimated breeding values ranged between 0.56 and 0.92.

**Conclusions:**

Results showed the viability of PLSR for QTL analysis and estimating breeding values using markers.

## Background

Detection of genomic regions affecting traits is a goal in many genetic studies. Studies applying distinct methods for detection of these regions, called quantitative trait loci (QTL), have been described, ranging from single marker regression [1] to methods that enable to fit several markers simultaneously [2,3]. Simultaneously fitting all markers leads to more accurate detection of QTL compared to independent fitting of single markers in a regression model when there is linkage disequilibrium (LD) between the genomic regions that affect the trait but comes at the cost of increased computational requirements [2].

Partial least square regression (PLSR) is one method for simultaneously fitting multiple markers and was applied by Bjornstad et al. for detection of QTL [3]. An interesting characteristic of PLSR its straightforward application of to simultaneous analysis of data of multiple traits [3].

The objectives of this study were to use PLSR to search for QTL and to estimate breeding values in the dataset of the QTLMAS 2010 workshop.

## Material and methods

### Initial analyses

The data were analyzed to identify generation and gender effects. Furthermore, a bivariate animal model was fitted in ASREML [4] using the matrix of additive genetic relations as relative covariance matrix to estimate variance components.

### Marker based analyses

Let **X** be the genotype matrix; the number of rows is the number of individuals and the number of columns is the number of markers. Elements of **X** are 0, 1, or 2, according to the number of one of the two alleles for that marker in that individual. Let **Y** be the matrix of phenotypes; the number of rows is the number of individuals and the number of columns is the number of traits in the data (two in these data).

We describe our regression methods in the following sections, beginning with an analysis where each trait was regressed on each marker independently and continuing with PLSR.

#### Single marker regression

We used function lm of R [5] to regress each trait on each marker. In this analysis, we used the same model for both traits and ignored the non-normal distribution of the discrete trait. We fitted the following model to the data:

**y***_t_*= *µ* + **x***_m_b* + **e**;    (1)

**e** ~ *N*(0,**I**σ^2^ );

where **y***_t_* is the vector of phenotypes for trait t, *µ* is a mean, **x***_m_* is the vector of genotypes corresponding to marker m, b is the unknown regression coefficient of **y***_t_* on **x***_m_* and **e** is the vector of residuals. Inference was based on ANOVA applied to the fitted models.

#### Partial least square regression

In PLSR, matrices **X** and **Y** are decomposed into principal components and loadings:

**X** = **TW**′

**Y** = **UQ**′    (2)

where **T** and **U** are the matrices of scores and **W** and **Q** are the matrices of loadings [6]. PLSR places two conditions in the decomposition of **X** and **Y**. The first requires orthogonality of **W** and **Q** and the second requires maximal correlation between the columns of **T** and **U**[6]. After decomposition, **U** is regressed on **T** :

**U** = **TB** + **E**,    (3)

where **B** is an unknown matrix of regression coefficients and **E** is a matrix of residuals. We used matrix **B** to calculate the matrix of regression coefficients of the individual markers, :(4)

##### Fitting

We treated the data of both traits equally, without accounting for the non-normal nature of the discrete trait. Since PLSR using all marker loci (~ 10000) was impossible, we calculated the regression coefficients in two steps. First, we regressed the phenotype data on the markers in each chromosome using PLSR and obtained the empirical distributions of these regression coefficients by bootstrapping. Second, we selected the 1000 most significant markers for each trait, defining significance of a marker as the absolute value of its regression coefficient divided by its empirical standard error. Subsequently, we regressed the phenotype data on the selected markers using PLSR and recalculated their standard errors using bootstrapping.

We used the R-package pls [7] to fit, cross validate, and use the PLSR models.

##### Detecting QTL

Our method assumed that the variance explained by markers reaches a maximum in the neighborhood of a QTL. We used locally weighted regression [8] to estimate a smoothed curve through the standardized regression coefficients of the markers, calculated as ( is the estimated regression coefficient for that marker and  is its empirical standard error, obtained from bootstrapping). We calculated the first and second derivative of this smoothed curve to find local maxima of the curve and we considered these local maxima as QTL. We calculated the variance explained by each QTL as , where p is its MAF and  is its regression coefficient from in the single marker regression analyses.

##### Calculating EBV

We estimated breeding values for all individuals in the data using the regression coefficients for the markers in the second PLSR model. Estimated breeding values (EBV) were calculated as .

## Results

### Initial analyses

The initial analysis revealed a positive correlation between the traits. No signals of selection nor sex effects were detected in the data.

The results showed that both traits were heritable and genetically correlated. Heritability of the first trait was 0.53 (s.e. 0.06) and heritability of the second trait was 0.22 (s.e. 0.04). The phenotypic correlation was 0.25 (s.e. 0.03) and the genetic correlation was 0.66 (s.e. 0.09).

### Single marker regression

**Figure 1:**
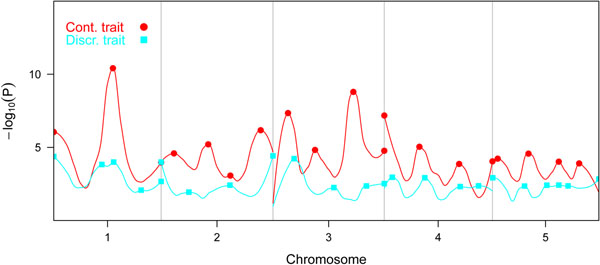
Smoothed curve of the -log(P) of the marker effects for the two traits, estimated using locally weighted regression.

Figure 1 shows the smoothed curve of the negative logarithm of the significances in the single marker analyses. QTL for the continuous trait were located on chromosomes 1 and 3 with smaller QTL on all chromosomes. The effects of QTL for the discrete trait were smaller compared to the the continuous trait with QTL on chromosomes 1, 2 and 3. Figure 1 suggests at least three pleiotropic QTL; one at approximately half the length of chromosome 1, one at the beginning of chromosome 3 and another at approximately 0.25 the length of chromosome 4.

### Partial least square regression with bootstrapping

**Figure 2:**
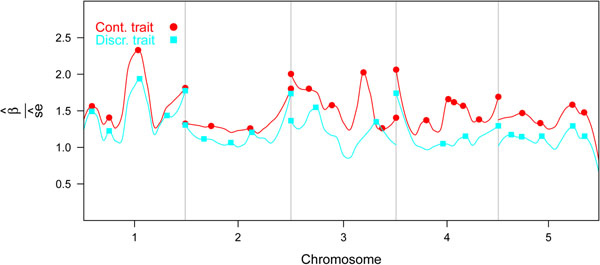
Smoothed curve of standardized regression coefficients of individual markers estimated with PLSR, estimated using locally weighted regression.

Figure 2 shows the smoothed curves through standardized regression coefficients of the PLSR models. Important QTL for the continuous trait were located on chromosomes 1, 3 and 4. The curves of chromosome 2 were remarkably flat compared to the results in Figure 1. QTL with an important effect on both traits were located on chromosomes 1, 3 and 4.

Local maxima identified in Figure 2 identified QTL, which are reported in Table 1 with regression coefficients and variance. The variance of QTL, expressed as a proportion of the total genetic variance, was small for both traits. The largest QTL for the continuous trait was located at position 1058 of chromosome 1 and explained 6.8% of the genetic variance, the largest QTL for the discrete trait was located at position 1977 of chromosome 2 and explained 4.3% of the genetic variance (Table 1). Based on this table, pleiotropic QTL were located between positions 156 and 159 and positions 494 and 495 on chromosome 1, between position 3242 and 3274 on chromosome 2, and between position 8890 and 8920 on chromosome 5 because these intervals harbored QTL affecting both traits (Table 1).

**Table 1:** Estimated regression coefficients and approximate standard error for the most significant markers in of the second PLSR model. The highlighted cell contain a regression coefficient which was considered most significant, the other cells contain the less significant regression coefficients.

marker		Cont. trait		Discr. trait	
		
	MAF				
**Chrom. 1**					
156	0.46	2.9920	0.0817	**0.0612**	**0.0405**
159	0.39	**-2.3459**	**0.0479**	-0.0654	0.0442
494	0.48	**0.3242**	**0.0010**	-0.0369	0.0148
495	0.06	-1.4611	0.0046	**0.0672**	**0.0116**
1058	0.31	**4.1433**	**0.1355**	0.0426	0.0170
1087	0.45	-0.4640	0.0020	**-0.0397**	**0.0170**
1621	0.41	0.2338	0.0005	**0.0108**	**0.0012**
1976	0.29	**-0.0818**	**0.0001**	**0.0336**	**0.0102**

**Chrom. 2**					
1977	0.37	**-0.3898**	**0.0013**	**-0.0924**	**0.0866**
2340	0.42	2.8491	0.0724	**0.0598**	**0.0378**
2481	0.31	**1.0574**	**0.0088**	0.0434	0.0175
2864	0.27	1.2254	0.0107	**0.0320**	**0.0087**
3242	0.09	**0.3485**	**0.0004**	-0.0043	0.0001
3274	0.31	1.3028	0.0134	**0.0867**	**0.0703**
4034	0.17	**1.5167**	**0.0119**	**0.0572**	**0.0200**

**Chrom. 3**					
4035	0.31	**-1.6002**	**0.0200**	**-0.0150**	**0.0021**
4384	0.37	**-1.3395**	**0.0153**	-0.0650	0.0427
4519	0.40	-0.1875	0.0003	**-0.0489**	**0.0249**
4832	0.47	**-1.7533**	**0.0281**	-0.0576	0.0360
5447	0.45	**1.9137**	**0.0333**	0.0062	0.0004
5695	0.46	-1.2392	0.0140	**0.0278**	**0.0084**
5811	0.38	**-2.1743**	**0.0407**	-0.0930	0.0883
6082	0.28	**0.6369**	**0.0030**	0.0123	0.0013

**Chrom. 4**					
6083	0.02	**3.1419**	**0.0078**	**0.0192**	**0.0003**
6671	0.46	**-1.4765**	**0.0199**	0.0126	0.0017
6995	0.06	5.3177	0.0585	**0.1009**	**0.0250**
7099	0.41	**-0.9055**	**0.0073**	-0.0352	0.0131
7209	0.46	**-0.3541**	**0.0011**	0.0010	0.0000
7386	0.39	**-0.4365**	**0.0017**	0.0109	0.0012
7433	0.46	0.4793	0.0021	**0.0590**	**0.0377**
7697	0.37	**-0.1112**	**0.0001**	0.0500	0.0252
8073	0.22	**2.1894**	**0.0300**	**0.0117**	**0.0010**

**Chrom. 5**					
8324	0.44	1.5840	0.0226	**-0.0076**	**0.0006**
8528	0.39	1.1567	0.0117	**-0.0035**	**0.0001**
8538	0.42	**1.0233**	**0.0093**	0.0027	0.0001
8890	0.35	**0.4145**	**0.0014**	0.0404	0.0162
8920	0.21	-0.1872	0.0002	**0.0565**	**0.0233**
9514	0.13	**-2.6520**	**0.0283**	0.0186	0.0017
9523	0.44	1.2587	0.0143	**0.0274**	**0.0080**
9744	0.21	**-1.1246**	**0.0078**	0.0487	0.0173
9754	0.16	-0.805510	0.0032	**-0.0094**	**0.0005**

The correlations between the EBV and the phenotypes and between the EBV and the true breeding values (accuracies of EBV) are displayed in Table 2. All correlations decreased from generation 0 to generation 3 for unknown reasons. The correlations in generation 4 where lower than in the previous generations because these individuals were not used to fit the model. The correlations between the phenotypes and the EBV where highest in the continuous trait but the correlations between the true breeding values and the EBV were highest in the discrete trait (Table 2).

**Table 2:** Correlations between estimated breeding values (EBV) and phenotypes (P) or true breeding values (TBV) of the continuous and discrete trait of individuals in simulated generations zero to three. TBV of all individuals and phenotypes of individuals in generation 4 were only used to evaluate the correlations but were not used to fit the models.

Trait	0	1	2	3	4
**Cont. trait**					
r(P,EBV)	0.70	0.70	0.68	0.69	**0.52**
r(TBV,EBV)	0.79	0.73	0.66	0.69	**0.56**

**Discr. trait**					
r(P,EBV)	0.64	0.63	0.55	0.55	**0.37**
r(TBV,EBV)	0.92	0.90	0.82	0.79	**0.72**

## Discussion and conclusions

We used a non conventional method to infer QTL, based on finding local maxima of a smoothed curves traced through the QTL probabilities (in the single marker regression) and through the standardized regression coefficients (in PLSR). This method assumed that a QTL will appear as a local maximum in the smoothed curves. An advantage of this method over methods that concentrate on profiles of single markers is that it combines evidence provided by a series of markers in the proximity of QTL. A disadvantage is that it does not provide a quantitative test statistic to statistically test for the presence of QTL.

Comparing the QTL detected with our method to true QTL locations revealed differences and similarities. We detected QTL on chromosome 5 while no QTL were simulated on this chromosome. This false detection is inherent to our method since detection was only based on local maxima in the curves. The method suggested many pleiotropic QTL and agreed with the truth, because the majority of the QTL were pleotropic.

We used single marker regression to estimate the variance of individual QTL because we expected that PLSR would underestimate the regression coefficients of QTL in LD with many markers. A disadvantage of this could be biased regression coefficients of QTL in LD with other QTL [2].

The correlations between EBV and true breeding values of individuals in generations 0 to 3 agreed with the correlations of EBV calculated in the studies of Meuwissen et al. [2]. Avoiding the need to preselect markers might lead to higher correlations for the nongenotyped individuals and the method Chun and Keles [9] might be an interesting alternative.

## Competing interests

The authors declare no competing interests.

## Authors’ contributions

AC analysed the data and wrote the manuscript. MPLC was involved in the discussion and conclusion of the manuscript.
